# A New Interaction Force Model of Gold Nanorods Derived by Molecular Dynamics Simulation

**DOI:** 10.3390/nano10071293

**Published:** 2020-07-01

**Authors:** Pan Yang, Qinghua Zeng, Kejun Dong, Haiping Zhu, Aibing Yu

**Affiliations:** 1 School of Engineering, Western Sydney University, Locked Bag 1797, Penrith, NSW 2751, Australia; pan.yang@westernsydney.edu.au (P.Y.); kejun.dong@westernsydney.edu.au (K.D.); h.zhu@westernsydney.edu.au (H.Z); 2 Centre for Infrastructure Engineering, Western Sydney University, Locked Bag 1797, Penrith, NSW 2751, Australia; 3 Department of Chemical Engineering, Monash University, Clayton, VIC 3800, Australia; Aibing.Yu@Monash.edu.au

**Keywords:** gold nanorods, interaction force model, molecular dynamics simulation, orientation configurations

## Abstract

Interactions between nanoparticles is one of the key factors governing their assembly for ordered structures. Understanding such interactions between non-spherical nanoparticles and developing a quantitative force model are critical to achieving the ordered structures for various applications. In the present study, the non-contact interactions of two identical gold nanorods (AuNRs) with different aspect ratios have been studied by molecular dynamics simulation. A new interaction potential and force model for two nanorods approaching side-by-side has been proposed as a function of particle surface separation and their relative orientation. In addition, the interaction potentials of two nanorods approaching in other typical orientation configurations (i.e., crossed, head-to-head and head-to-side) have also been investigated.

## 1. Introduction

Interparticle interactions are common and exist in all length scales from planets, powders, molecules to atoms, either attractive or repulsive [1,2,3]. It has been well recognised that their interaction forces (e.g., van der Waals attraction, Born repulsion, electrostatic interaction, gravitation, hydrophobic, capillary) play a critical role in governing and controlling their various dynamic behaviours as well as the formation of versatile and ordered structures, such as suspending, aggregating, packing, and flowing [4,5,6,7,8]. A quantitative study will allow the prediction of particle self-assembly for ordered structures and functionalised materials with significant commercial values [9,10].

Theoretically, interparticle interactions and force models for particles at atomic scale (<10^−9^ m) and micro-scale (>10^−6^ m) have been well established and expressed in different formats of force equations such as Lennard-Jones, Buckingham, Hamaker, and DLVO (Derjaguin–Landau–Verwey–Overbeek) [11,12,13,14]. Yet, the precise and quantitative calculation of interactions between nanoparticles (1 to 100 nm) is extremely challenging and still not well understood because of their surface effect and discrete atomic structure [15,16]. 

Research efforts have been made to measure the interparticle forces by atomic force microscopy (AFM), scanning tunnel microscopy (STM) or combined method. However, there are some limitations in these experimental methods such as the requirement of sample pre-treatment, the complexity of apparatus operation, and the precision of cantilever [17,18]. The experimental conditions may also vary significantly and are hard to control. On the other hand, with the rapid development of computational power and advanced algorithms, numerical simulations could provide an alternative way and overcome such drawbacks. 

Over the past years, various molecular modelling and simulation techniques have been applied to study and quantify the interactions between nanoparticles, including ab initio, density functional theory (DFT), Monte Carlo, and molecular dynamics (MD) simulation [19,20,21,22]. Zeng et al. [15] explored the interaction forces on SiO_2_ nanospheres of different size (i.e., 2, 5, 6.84 and 9 nm) by MD simulation and reported that there is a considerable deviation of interaction forces when applying conventional Hamaker’s approach to nanoparticles. Later, Sun et al. [16,23,24,25,26] performed MD simulations to calculate the interaction forces of different nanospheres and quasi nanospheres (e.g., silica, silicon, carbon) and compared their results with some classical approximations. They have successfully developed some equations to delineate the interaction force of nanoparticles (<10 nm). Recently, a new approach has been reported by Yang et al. to quickly quantify the interaction potentials and forces between gold nanospheres and applied to nanospheres with a diameter of up to 20 nm [27]. They developed an exponential force equation for gold nanospheres with the values of force constants between those of atomic and colloidal particles.

However, quantitative calculation between non-spherical nanoparticles (e.g., nanorod, nanowire, and nanoellipsoid) is much more complicated than that of nanospheres and has not well been explored. This is due to the complexity of their geometry (i.e., particle shape) and uncertainty of their surface effect. In addition, apart from the particle separation reported in the previous studies on nanospheres, the factor of relative orientation between two non-spherical nanoparticles could play a significant role in their interaction forces, which has not yet been well investigated quantitatively.

Gold nanoparticles (AuNPs) are one of most important and extensively studied materials since the nanotechnology emerged in the 1990s. They have demonstrated extraordinary optical, electromagnetic, biocompatible, and chemical properties. This has led to a variety of technological and innovative applications of gold nanomaterials permeating in many fields, such as semiconductors, bioimaging, catalysts, drug delivery, and cancer therapy [28,29,30,31]. Specifically, gold nanorods (AuNRs) have shown great potentials for various commercial and industrial applications. Moreover, under proper control and conditions, AuNRs can be assembled into 1D, 2D and 3D functionalised nanomaterials. Many of such assembly processes are dominated by the interaction forces between AuNRs. Yet, an accurate and quantitative force model is critical to exploring such promising potentials [32,33].

In this study, MD simulations have been performed to explore the interaction potentials and establish a new force model for two identical AuNRs with a diameter of 2 nm and different aspect ratios (i.e., length to diameter, L/D). Specifically, during the simulation, two AuNRs are allowed to have a translational movement and a relatively rotational movement to each other. This paper is outlined as follows. Section 2 briefly introduces the simulation methods and conditions. Section 3 presents and discusses the simulation results of AuNRs approaching side-by-side, the dependence of interparticle interactions on various factors (e.g., particle separation, relative orientation, aspect ratio). A new interaction force model of AuNRs will be established via data fitting of our simulation results. The interaction energies of four typical configurations of AuNRs (i.e., side-by-side, crossed, head-to-side and head-to-head) have also been revealed. The conclusions have been summarised in the last Section.

## 2. Simulation Methods and Conditions

All MD simulations are carried out by Forcite module in Materials Studio package (version 7.0, BIOVIA, San Diego, CA, USA) [34]. The state-of-the-art COMPASS II force field has been employed to calculate the interactions between AuNRs. This force field enables a precise and full description of the interactions of all atoms in the system, including bonded interactions (i.e., stretching, bending, torsion and angle inversion) and non-bonded interactions (i.e., van der Waals, electrostatic and hydrogen bond) [35]. For a system of two nanoparticles (i.e., NP1 and NP2) at a given separation *d*, the interparticle interactions $\left(E_{Inter}\right)$ can be expressed as,

(1) $$E_{Inter\;}\left(d\right)=E_{Total}-E_{NP1}-E_{NP2}$$

where $E_{Total}$ is the potentials of the system of combined nanoparticles, and $E_{NP1}$ and $E_{NP2}$ are the potentials of isolated nanoparticles, NP1 and NP2, respectively. Once the interparticle potentials have been determined at different particle surface separations, the interparticle force upon surface separation can be derived and written as the gradient of interparticle potentials with respect to the particle surface separation and written as,

(2) $$F_{Inter\;}\left(d\right)=-\frac{\partial E_{Inter\;}\left(d\right)}{\partial \left(d\right)}$$

The MD simulation method is similar to our previous work on gold nanosphere [29]. There are some significant changes on the simulation process to allow the study of the dependence of AuNRs interactions on their relative orientation. The major simulation procedures and distinctions for non-spherical AuNRs are outlined as follows.

(1) An atomic model of AuNRs with a diameter of 2 nm and a specific aspect ratio (e.g., L/D = 4) is built from a gold crystal supercell. Then, two AuNRs are initially placed with a surface separation *d* of near zero interaction force (e.g., 0.8 nm in our cases) under a side-by-side orientation unless specified. For nanoparticle systems, gravitational force can be ignored. Our model of two AuNRs is in vacuum condition although nanorod systems mainly present in aqueous solutions. The effect of solution on the interaction forces will be investigated in our future work. Atomic models of AuNRs with an aspect ratio from 4 to 7 have been studied.

(2) Prior to a MD simulation, the system of two AuNRs was fully relaxed at 289 K with NVT (i.e., constant number of atoms, constant volume, and constant temperature) ensembles and time step of 1 fs, aiming to obtain an equilibrium configuration with minimal energy.

(3) MD simulations were then performed on the geometry optimized system of AuNRs like the approach we used for the system of two gold nanospheres [27]. A small and arbitrary external force (e.g., 0.139 nN) with same magnitude but opposite direction (along X axis) was applied to each AuNR. The external force, with its magnitude depending on the mass of AuNRs, allows the generation of an initial but opposite velocity for both AuNRs to approach each other during the simulation. Such force will reduce significantly the simulation time since there is a near zero interaction force at their initial separation and it is almost impossible for them to approach each other. The applied external force was then ceased at a certain time. This ensures that the remaining dynamic process is smooth enough and dominantly controlled by the interparticle interactions. Meanwhile, it also minimises the potential effect of the external force on the results. Finally, the MD simulation will stop when a given surface separation between the AuNRs has been reached. This is because the present work is to quantify the non-contact interaction forces between AuNRs. Also, we observed that deformation occurs in the AuNRs when their surface separation is within a couple of bond lengths, which does not represent the original shape of AuNRs.

(4) During the simulation, one AuNR (e.g., NP2) was forced to rotate continuously at a certain degree within a given time interval (e.g., 1 degree in every interval of 0.01 ps) by its mass centre along the X axis. Therefore, the AuNRs could achieve both the translational and rotational movements. Such dynamic behaviour was common but rarely applied in the previous studies. In addition, the simulation results (e.g., bonded and non-bonded interactions, velocity, coordinates, rotation angle and surface separation) are recorded at a frequency of 100 steps in an output trajectory during the simulation. Last, it is worth mentioning that the force field assigns zero charge to gold atoms. Thus, the electrostatic interaction between AuNRs can be ignored.

(5) Data analysis

After the MD simulation, data analysis is made to the interparticle energies, surface separation and rotation angle of the given system of AuNRs. Specifically, the data of interaction energy potential (Z axis) between two AuNRs over surface separation (X axis) and rotation angle (Y axis) are plotted in a 3D graph. The projections of energy onto 2D graph are also presented. Then, such data are fitted to achieve a quantitative equation to describe the potential as a function of surface separation and rotation angle. Finally, a quantitative force model can be derived by Equation (2) as the gradient of energy potentials and expressed as a function of surface separation and rotation angle of two AuNRs.

## 3. Results and Discussions

### 3.1. Models and Dynamic Collision of AuNRs

**Figure 1:**
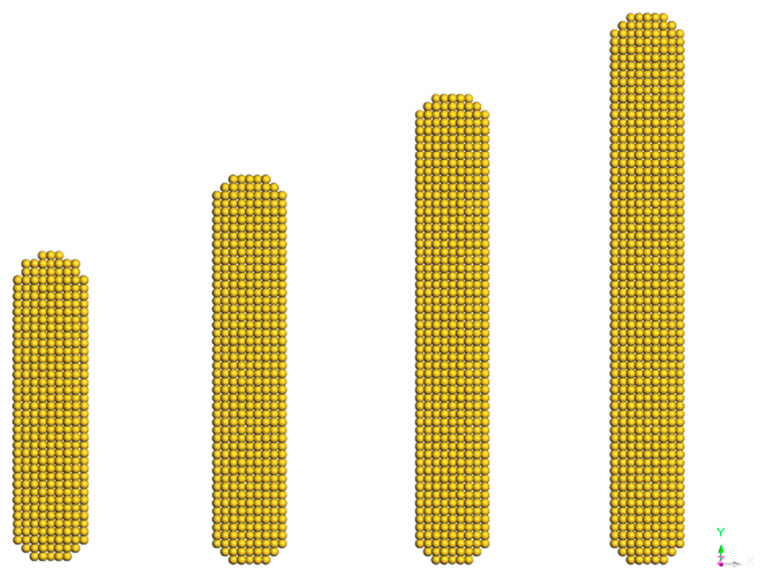
Illustration of a single gold nanorod with a diameter of 2 nm and an aspect ratio (L/D) of 4, 5, 6, and 7 (from left to right), respectively.

Figure 1 shows a series of AuNRs of different aspect ratios from 4 to 7 with a diameter of 2 nm. And the numbers of atoms of them are 1255, 1588, 1921, and 2266, respectively. To study their interactions, two identical AuNRs with a certain surface separation (e.g., 0.8 nm) are placed and allowed to approach towards each other under an applied external force (0.139 nN) with the same magnitude but an opposite direction. The initial velocities generated under applied external force of AuNRs with different aspect ratios are about 0.3 m/s. In particular, one of the AuNRs (e.g., right-hand one) will be assigned an additional time-dependent rotation action along X axis by its mass centre at every 0.01 ps.

**Figure 2:**
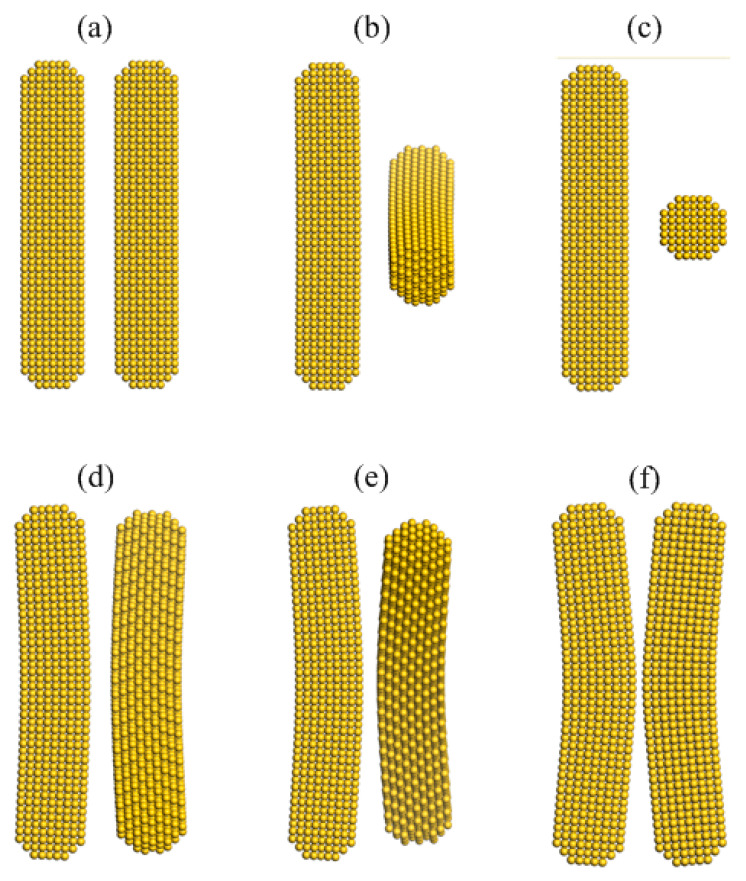
Snapshots of a pairwise identical AuNRs side-by-side with a diameter of 2 nm and an aspect ratio of 5 during their dynamics interacting process: (**a**) 0 ps, (**b**) 0.89 ps, (**c**) 10.1 ps, (**d**) 14.6 ps, (**e**) 20 ps, and (**f**) 21.59 ps.

Figure 2 demonstrates some typical snapshots from the dynamic interaction process of AuNRs with a diameter of 2 nm and an aspect ratio of 5 (i.e., L/D = 5) from an initial surface separation to a near contact position (Figure 2a–f). Figure 2b shows a crossed configuration of AuNRs when rotation angle (ϴ) equals to π/2. A slight deformation of AuNRs is observed when the surface separation further reduces to 0.65 nm (Figure 2d), leading to the change of the geometry and rearrangements of surface atoms, especially at the central area of interacting AuNR surface. Such geometry deformation increases with simulation time and the reduction of surface separation. Figure 2f indicates that AuNRs are at the point of jump-to-contact and will almost be overlapping with surface separation of 0.38 nm as a result of such geometry bending. Likewise, the geometry deformation also occurs in other sizes of AuNRs during their side-by-side collision, which emerges at the surface separation of about 0.61 nm (L/D = 4), 0.69 (L/D = 6) and 0.71 (L/D = 7) nm, respectively. This deformation and facture phenomena have been previously investigated and reported by numerical methods when studying the discrete particles [36,37,38]. Such earlier stage of deformation with the increase in aspect ratio could be explained as the result of an increase in interacting surface areas at the same surface separation during their dynamic approaching process.

### 3.2. Interparticle Potentials of AuNRs with Different Aspect Ratios

As we mentioned earlier, interaction potentials for particles at atomic scale (<10^−9^ m) and micro-scale (>10^−6^ m) have been well established and predicted with different classical formulas or empirical approximations, therefore, it is critical to fulfil the gap for nanoparticles that have significant surface effect. In our previous study, a quantitative model with coefficients of exponential order *m* and constant A was developed to describe the interparticle potentials of gold nanospheres with a diameter of up to 20 nm [27].

It is well known that the interactions between nanoparticles are highly dependent on their shape (geometry) and size. In addition, the non-spherical shape of AuNRs makes it much more complicated to quantify their interactions because of the asymmetry of their geometry and uncertainty of bonding conditions of surface atoms.

**Figure 3:**
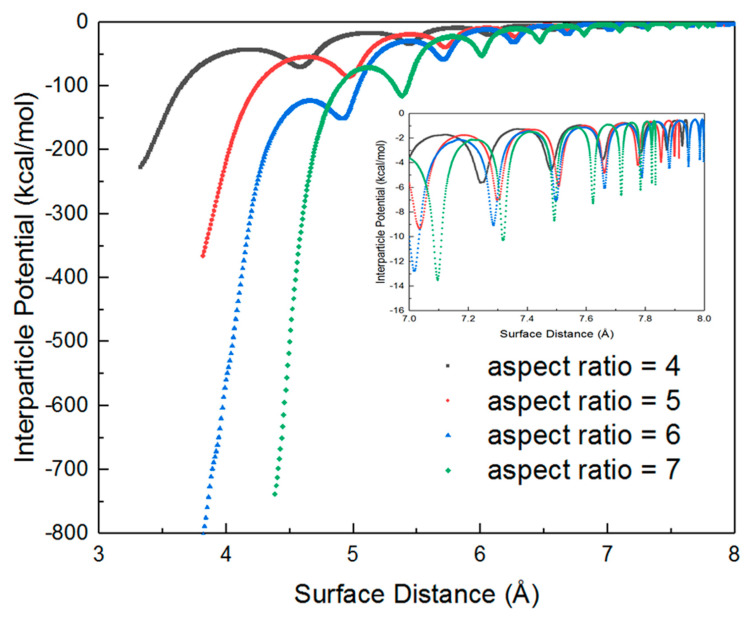
Interparticle potential as a function of surface separation of AuNRs with a diameter of 2 nm and different aspect ratios (L/D): 4 (black), 5 (red), 6 (blue), and 7 (green); the insert is the interparticle potential as a function of surface separation at very early stage (i.e., 0.7–0.8 nm).

In the present study, only non-contact interactions at a surface separation longer than about two bond lengths of gold will be considered because deformation of AuNRs occurs at a shorter surface separation. Figure 3 represents the dependence of interparticle potentials as a function of surface separation between two identical AuNRs with different aspect ratios. The interparticle potentials are negative, which means an attractive interaction. Moreover, the magnitude of potentials increases with the decrease of surface separation following an exponential trend, which agrees with the results of gold nanospheres in our previous study. However, it is interesting to observe that there is a deformed sinusoidal pattern along such an exponential trend, which coincides with the period of rotation angle during the dynamic interacting process. Such deformed sinusoidal pattern is due to the increase in the amplitude (e.g., interaction potential), resulting from the decrease in surface separation within a period of rotation. Moreover, the magnitude of potentials is significantly affected by the AuNR aspect ratio at shorter surface separation. It increases more rapidly with the decrease in surface separation for AuNRs with higher aspect ratios. Yet, all AuNRs in the present study have a similar surface distance at which interparticle potentials reach a near zero value.

**Figure 4:**
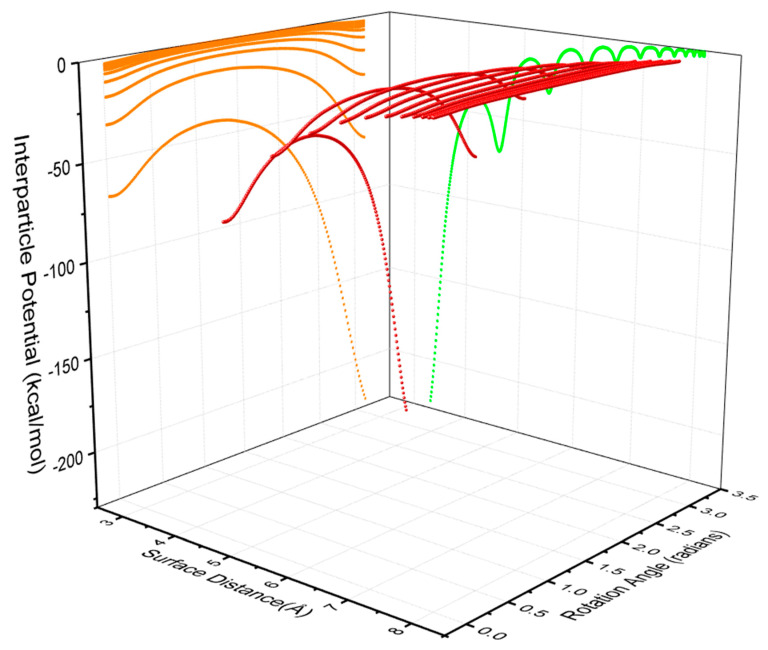
3D illustration of interparticle potential (in red) as a function of surface separation and rotation angle in radians of a pairwise AuNRs with a diameter of 2 nm and aspect ratio of 4. The projected curves indicate the dependence of potential on rotation angle (in amber) and surface separation (in green), respectively.

Specifically, Figure 4 shows a 3D image of the dependence of interparticle potentials on both surface separation and rotation angle. It is observed that the magnitude of potentials increases with the decrease in surfaces separation and the angle of orientation (i.e., the absolute intersection angle between the long axis of both AuNRs with a value of 0 to 90 degree). In other words, given a period of 180 degrees, the interaction potential of two AuNRs will reduce from the value at 0 degree to the minimal value at 90 degree (π/2). The latter corresponds to a crossed configuration of two AuNRs (Figure 2b). Then, it will increase again from the position of 90 degrees (π/2) to the maximal value at 180 degrees (π). Such a change pattern corresponds to a half period of a sinusoidal function, yet it is asymmetrical. This is because the increase of interparticle potentials upon the surface separation was not proportional within a period of rotation as shown by the amber line in Figure 4.

**Figure 5:**
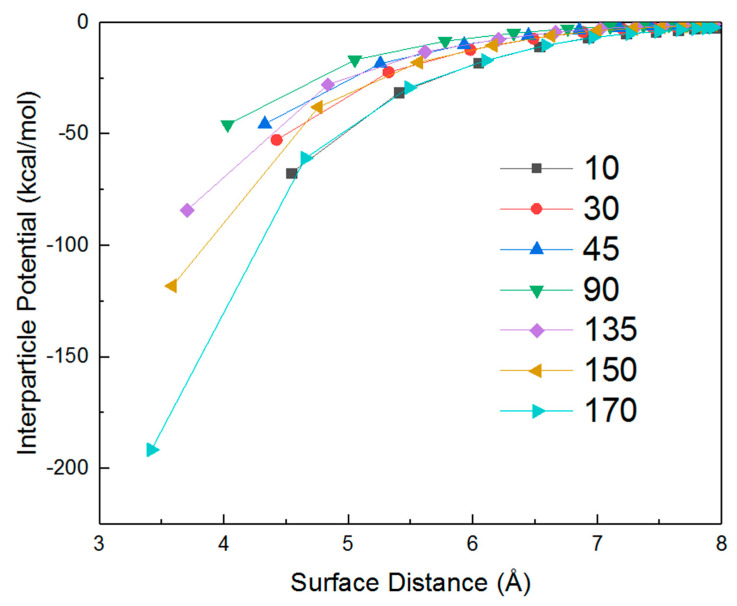
Interparticle potential as a function of surface separation of a pairwise AuNRs with a diameter of 2 nm and aspect ratio of 4 under a given relative rotation angle.

The above pattern of potential change also coincides with the change of an effectively interacting surface (the level of overlap of both AuNRs viewed from X axis) within a period of AuNR rotation. To further examine the relationship between potential and effective interaction surface, we have plotted the potential over surface separation at several specific angles of rotation for AuNRs with an aspect ratio of 4 (Figure 5). During the rotation of one AuNR, their relative configuration varies with the time as well as rotation angle. Meanwhile, the interacting surface area can be symmetrically separated at 90 degrees within the range of 0 to 180 degrees. For example, Figure 5 indicates the curve trend at 30 degrees is similar to that of 150 degrees. Similarly, the curve at 45 degrees has a close tendency with the curve of 135 degrees. At a given separation, the minimal value of interparticle potentials at 90 degrees represents a crossed configuration between two AuNRs and the lowest effectively interacting surface.

### 3.3. Interparticle Potential and Force Models of AuNRs with Different Aspect Ratios

**Figure 6:**
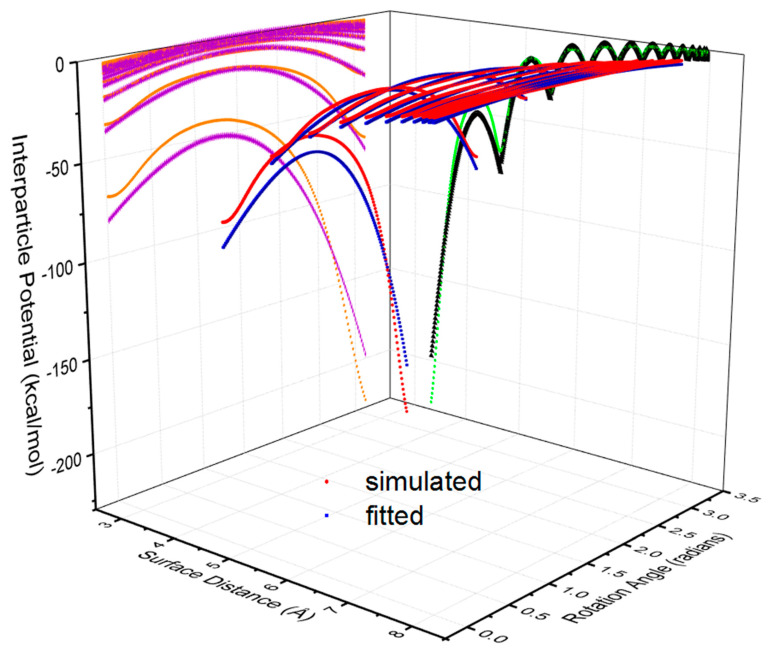
3D image of simulated (in red) and fitted (in blue) curves of interparticle potentials of AuNRs with a diameter of 2 nm and aspect ratio of 4 with respect to surface separation and rotation angle. Their projected curves indicate the dependence of potential on rotation angle (in orange and purple) and surface separation (in green and black), respectively.

In the present study, the only existing interparticle interactions between AuNRs are non-bonded van der Waals force as there is no charge assigned on gold atoms. Such vdW force is relatively weak and generally ignored at macro-scale. Yet, it becomes critically significant when particle size goes down to nanoscale, particularly in nanoparticles. For a pairwise AuNRs, their interparticle potential is actually the summation of van der Waals interactions of all pairwise atoms. Figure 6 represents both simulated data and fitting results for AuNRs with a diameter of 2 nm and aspect ratio of 4. The following Equation has been developed as the interaction potential model for such AuNRs with their constants shown in Table 1.

(3) $$E_{Inter}^{MD}=\frac{Eo+A01*d+B01*\theta +B02*\left(\theta^{2}\right)+B03*\left(\theta^{3}\right)}{1+A1*d+A2*\left(d^{2}\right)+A3*\left(d^{3}\right)}$$

**Figure 7:**
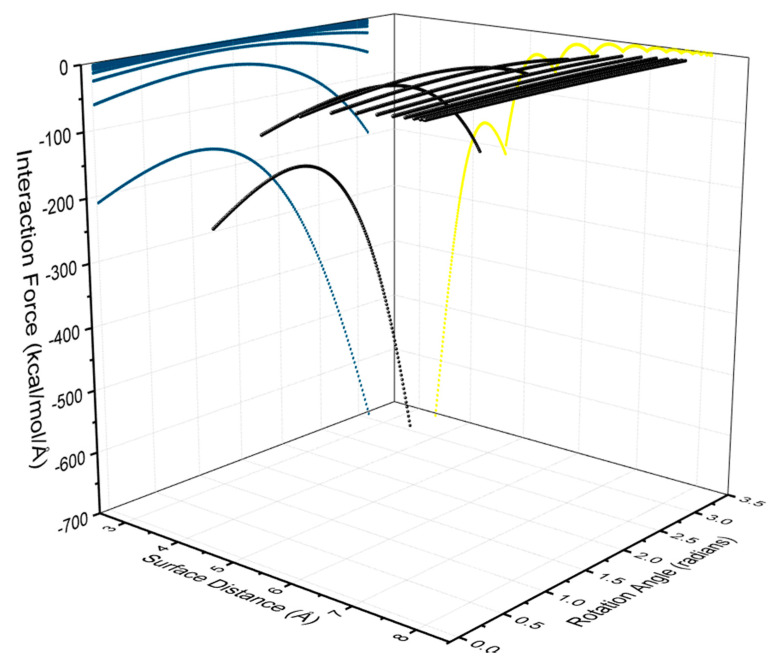
3D image of interaction force (in black) of AuNRs with a diameter of 2 nm and aspect ratio of 4 with respect to surface separation and rotation angle. The projected curves indicate the dependence of force (in yellow) on surface separation and torque (in blue) on rotation angle, respectively.

From the above interaction potential model, the interparticle force of AuNRs can be determined as a result of the differential coefficients from their interparticle potential model in Equation (3). Specifically, the force and torque models can be achieved as the negative of the partial derivative of potential energy with respect to surface separation and rotation angle, respectively. Such models have taken into account the relative rotation in addition to surface separation between two AuNRs. The obtained interaction force model is presented in Figure 7, with the projected curves indicating the dependence of force and torque on rotation angle and surface separation. This allows us to investigate the behaviors and dynamics of nanoparticles at a nano-scale level while other established models such as Gay-Berne allow the study of molecular systems [39,40,41].

(4) $$F_{Inter}^{MD}\left(\theta \right)=-\frac{\partial E_{Inter\;}\left(\theta \right)}{\partial \left(\theta \right)}$$

(5) $$F_{Inter}^{MD}\left(d\right)=-\frac{\partial E_{Inter\;}\left(d\right)}{\partial \left(d\right)}$$

### 3.4. Interparticle Interactions of AuNRs with Different Configurations

**Figure 8:**
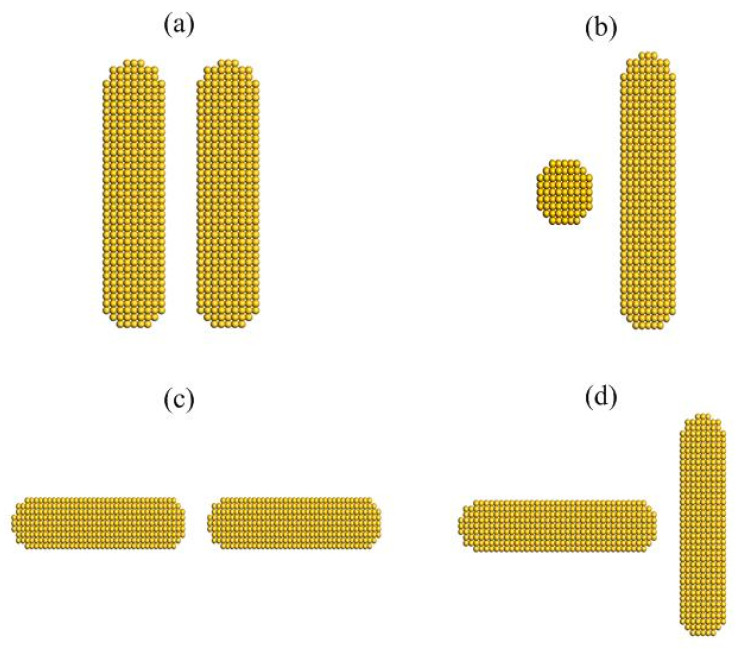
Typical configurations of a pairwise AuNRs with a diameter of 2 nm and aspect ratio of 4: (**a**) side-by-side, (**b**) crossed, (**c**) head-to-head and (**d**) head-to-side.

In addition to side-by-side configuration, there are several other typical configurations or orientations in regular non-spherical particles like wires, rods and ellipsoids. To examine the interparticle interactions of other orientations, MD simulations have been performed on the other three typical configurations (i.e., crossed, head-to-head and head-to-side) as shown in Figure 8. In each configuration, an external force is applied to two identical AuNRs in the same way as side-by-side configuration. In addition, the right-hand nanorod will be forced to rotate (along X axis) at the same rate as that in side-by-side MD simulations.

**Figure 9:**
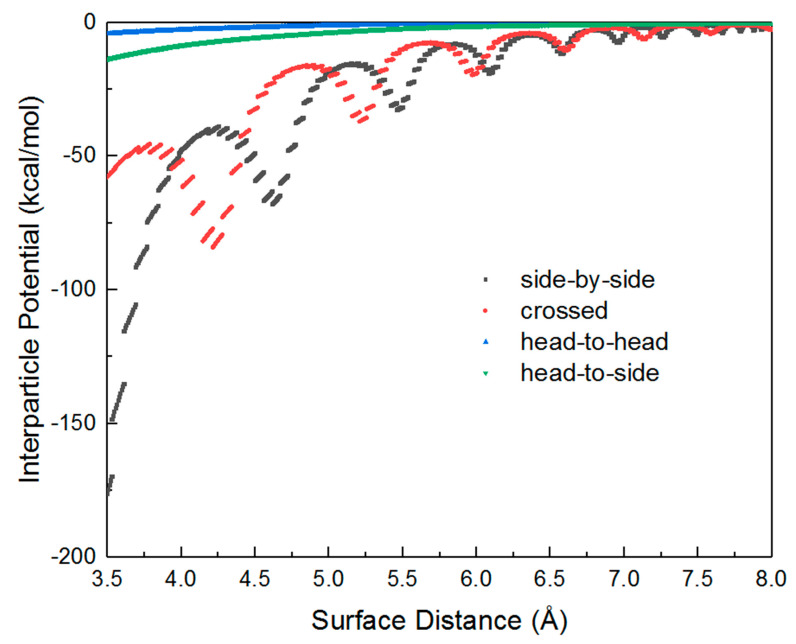
Interparticle potential as a function of surface separation of a pairwise AuNRs with a diameter of 2 nm and aspect ratio of 4 of four typical configurations: side-by-side (in black), crossed (in red), head-to-head (in green), and head-to-side (in blue).

Figure 9 shows that the interparticle potentials of crossed AuNRs have the same pattern as side-by-side configuration, yet have an almost π/2 phase shift compared with that of side-by-side case. This is because side-by-side configuration changes into crossed configuration after a rotation angle of π/2 as indicated above. Moreover, the interparticle potentials of head-to-side and head-to-head configurations are different from side-by-side and crossed configurations. There is no impact with rotation. This is because the rotation of the AuNR aligns with the X axis. In addition, the magnitude of potentials in both configurations are much lower than that of the side-by-side and crossed one, since their interacting surface is less and almost identical. Therefore, their interparticle potential is more likely to be dependent on the surface separation and can be expressed as exponential models similar to those of nanospheres.

## 4. Conclusions

Understanding and quantifications of the interactions between nanoparticles is complicated but crucial to explore their dynamic behaviours for promising applications. In the present study, efforts have been made to unfold the underlying interactions between two identical gold nanorods. Such interparticle interactions have been determined by molecular dynamics simulations. It provides an effective and precise approach to quantitatively calculate the interparticle potentials of non-spherical nanoparticles, which is challenging to achieve experimentally. Specifically, an external force and a forced rotation have been applied to AuNRs during the MD simulations, and the following conclusion can be drawn from the present work:

(1). A quantitative interaction model has been developed for gold nanorods with a side-by-side configuration, which is expressed as a function of surface separation and relative orientation angle;

(2). The magnitude of interaction potentials in side-by-side configuration increases with the decrease of surface separation and orientation angle from 90 to 0 degree. Deformation of AuNRs has been observed at a close surface separation;

(3). The pattern of interparticle potentials for crossed configuration is similar to that of side-by-side configuration, but has a π/2 phase shift;

(4). The interparticle potentials of head-to-head and head-to-side configurations are independent on the rotation angle (along X axis) and their magnitude is much lower than that of side-by-side and crossed configurations due to the relatively less effective interacting surface area.

The developed interaction model can be applied to the discrete element method and investigate the assembly or packing of AuNRs. The present work also provides a way to design nanoparticles with controlled interparticle interactions for specific ordered structures and materials. Further work is under way to investigate the self-assembly of AuNRs and the effects of a solution and nanorods with different aspect ratios on their interaction forces.

## Figures and Tables

**Table 1:** Constants for the fitted interaction potential models of AuNRs with a diameter of 2 nm and different aspect ratios (L/D).

Aspect Ratio (L/D)	4	5	6	7
Eo	195.10	1157.96	13476.12	23103.31
A01	−13.85	−90.06	−1314.02	−2192.18
B01	−110.73	−602.08	−8091.66	−8719.83
B02	38.79	211.73	4505.65	2012.23
B03	−1.47	−8.49	−657.60	310.59
A1	−1.90	−8.74	−37.60	6.91
A2	0.75	4.07	20.67	12.74
A3	−0.10	−0.54	−2.99	−3.26
R^2^	0.99	0.99	0.99	0.99
